# A dual-view multi-resolution laparoscope for safer and more efficient minimally invasive surgery

**DOI:** 10.1038/s41598-022-23021-2

**Published:** 2022-11-02

**Authors:** Jeremy Katz, Hong Hua, Sangyoon Lee, Mike Nguyen, Allan Hamilton

**Affiliations:** 1 James C. Wyant College of Optical Sciences, The University of Arizona, 1630 E University Blvd., Tucson, AZ 85721 USA; 2 Department of Urology, Keck School of Medicine of USC, Los Angeles, CA USA; 3 Division of Neurosurgery, Department of Surgery, University of Arizona, Tucson, AZ USA

**Keywords:** Endoscopy, Biomedical engineering

## Abstract

Minimally invasive surgery (MIS) is limited in safety and efficiency by the hand-held nature and narrow fields of view of traditional laparoscopes. A multi-resolution foveated laparoscope (MRFL) was invented to address these concerns. The MRFL is a stationary dual-view imaging device with optical panning and zooming capabilities. It is designed to simultaneously capture and display a zoomed view and supplemental wide view of the surgical field. Optical zooming and panning capabilities facilitate repositioning of the zoomed view without physically moving the system. Additional MRFL features designed to improve safety and efficiency include its snub-nosed endoscope, tool-tip auto tracking, programmable focus profiles, unique selectable display modalities, foot pedal controls, and independently controlled surgeon and assistant displays. An MRFL prototype was constructed to demonstrate and test these features. Testing of the prototype validates its design architecture and confirms the functionality of its features. The current MRFL prototype functions adequately as a proof of concept, but the system features and performance require further improvement to be practical for clinical use.

## Introduction

Due to its numerous benefits, minimally invasive surgery (MIS) has become the standard for many procedures such as appendectomies and cholecystectomies^1,2^. Compared to traditional open surgery, MIS boasts shorter recovery times and hospital stays, as well as reductions in post-operative pain and medical costs^3,4^. These benefits have catalyzed the evolution of technology within the field of MIS and given rise to advancements such as laparo-endoscopic single-sight surgery, energized instruments, and robotic surgical systems. Current MIS technology, however, exhibits several inherent limitations.

First, state-of-the-art laparoscopic and robotic technologies limit situational awareness and pose safety concerns due to an inherent trade-off between instantaneous field of view (FOV) and image resolution^5^. To facilitate detailed close-up work, a commercial laparoscope typically exhibits a narrow FOV, which provides a “keyhole” view of the intervention site. When operating with a narrow view of the surgical field, it is possible for complications outside the FOV to go unnoticed, such as tissue and nerve trauma from instrument collisions and electrosurgical burn injuries^6–8^. Currently, this issue is addressed in both conventional laparoscopic surgery (CLS) and robotic surgery by advancement and withdraw of the laparoscope, which requires a trained assistant for CLS and reduces the efficiency of robotic surgeries. Physically advancing the laparoscope results in a closeup view of the surgical site, while withdrawing the laparoscope yields a wide view of the operating cavity. This technique offers the ability to see and avoid surrounding obstacles, such as tissue and instruments, while inserting and guiding tools to the surgical region of interest (ROI). However, instruments are swapped out frequently during MIS. Thus, it is inefficient for a surgeon to cease operating so that the laparoscope can be retracted to gain a wide view each time an instrument is introduced. Consequently, instruments are often inserted and guided blindly until they appear in the narrow camera view. During blind tool manipulation, there is an increased risk of collisions, direct coupling, or arcing occurring and going unnoticed^6,7,9,10^. Improving situational awareness during surgery may lessen the frequency of such complications and increase the likelihood of detection should they occur^8,11^.

**Figure 1:**
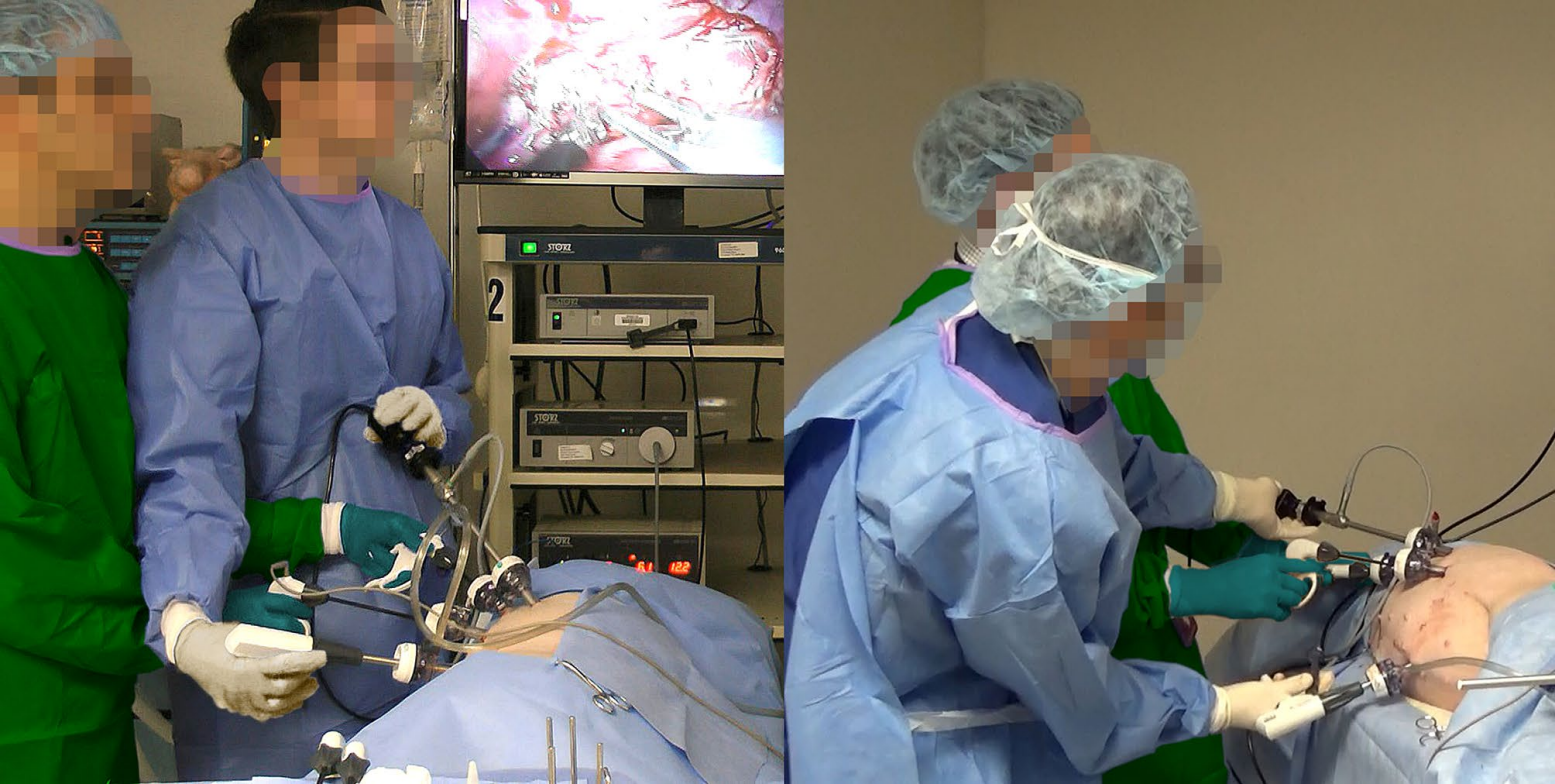
Candid photos of surgeon-assistant pairings performing porcine MIS with a standard laparoscope. Ergonomic posturing and ranges of motion are sacrificed to position the hand-held laparoscope at an appropriate viewing angle (note: gowns and gloves were re-colored using image processing software to ease differentiation between the limbs of the surgeons and assistants. Barring face pixilation, no additional image processing was performed).

Second, the efficiency of MIS is decreased by the non-stationary nature of traditional laparoscopes and its heavy reliance on the working dynamic between surgeon and assistant^12–15^. Though somewhat resolved by stabilized motion control in robotic surgery, these limitations are especially challenging in CLS where a trained assistant holds and maneuvers the camera for surgeries that typically last 75 to 99 min (though some last longer than 3 h)^16–18^. During a procedure, camera stability can decrease noticeably because of muscle fatigue^19^. Further, it is common for the surgeon and assistant to work over each other in awkward and ergonomically taxing positions to achieve adequate viewing angles of an ROI (see Fig. 1). Such positions exacerbate fatigue and limit range of motion, thereby slowing progress and increasing the risk of complications.

Three categories of related technologies have emerged to mitigate the limitations discussed above through real-time wide-FOV capture: augmented laparoscopes, custom imaging trocars, and intracorporeal vision networks. Augmented laparoscope solutions involve modifying existing laparoscope architectures with additional technology or devices, such as scanning prism assemblies^20,21^ or panomorph lenses^22^, to expand the viewable area. Custom imaging trocars (CITs), like the Enhanced Laparoscopic Vision System^23,24^, feature cameras and lights that capture large FOVs to supplement or replace traditional laparoscopes. To facilitate insertion through the abdominal wall, most CITs exhibit mechanical mechanisms, such as sliding tacks or folding arms^23–28^, for remote intracorporeal deployment. Intracorporeal vision networks (IVNs) are comprised of one^29^ or more^30–33^ stand-alone imaging devices that are mounted or placed inside the abdominal cavity. Most competing technologies that fall under these categories provide peripheral context via intracorporeal innovations that add significant setup time and complexity to MIS while only solving a fraction of its limitations. A full comparison between the limitations of these categories and the system proposed in this paper is presented in Supplementary Table S1.

A dual-view endoscope system that simultaneously captures a wide view and zoomed view has also been proposed^34,35^. The system features optical zoom and uses linear translation of a porro prism to shift the zoomed view image to different regions of the surgical field. Though promising, the dual-view endoscope is fundamentally limited by the porro prism shifting mechanism. Translational motion tends to be slower and less repeatable than rotational motion for high precision dual-axis applications. Further, the porro prism image shifting mechanism places size restrictions on the system and limits miniaturization.

We have been developing a novel multi-resolution foveated laparoscope (MRFL) and accompanying software to address both the safety and efficiency concerns outlined above. The MRFL system and software architectures improves upon the working principles of the dual-view endoscope with a more robust and precise scanning mechanism and additional features designed to improve MIS safety and efficiency. The evolution of the MRFL technology thus far has been reported in various technical papers that detail the design, enhancement, programming, and testing of its different subsystems and software features^36–44^. This paper provides a comprehensive overview of the current MRFL system and software features designed to improve MIS safety and efficiency and reports the preliminary testing and validation of these features.

## Methodologies

**Figure 2:**
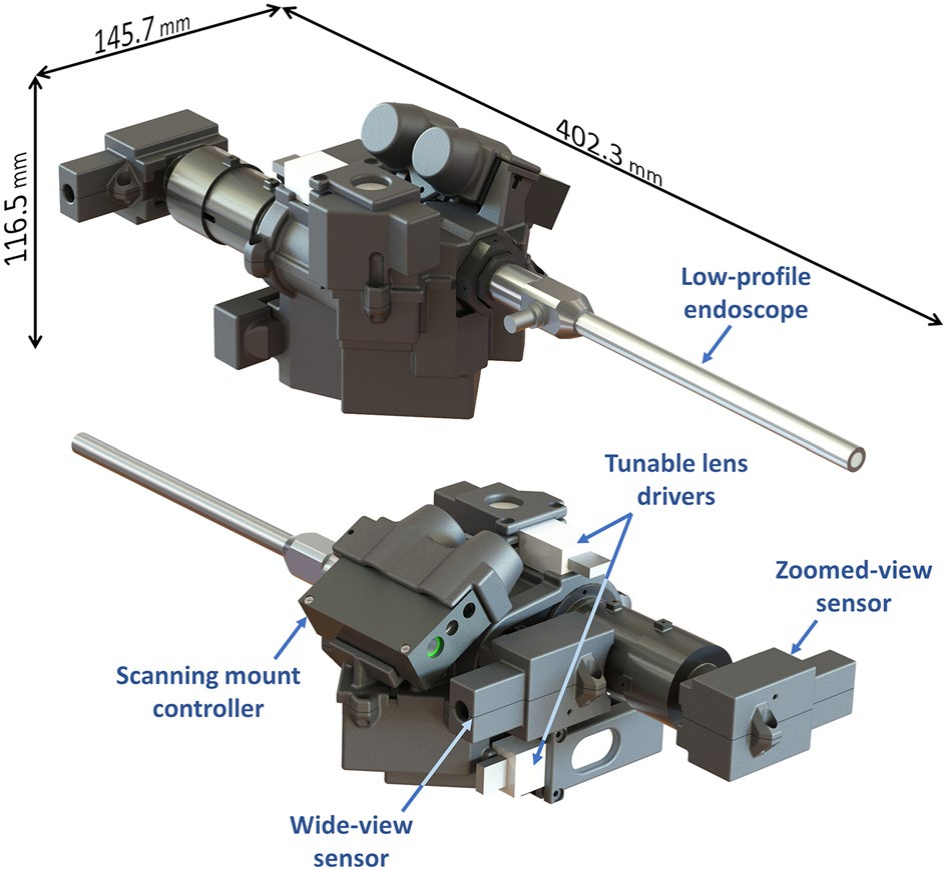
Overview of the current MRFL prototype. The system is a rail mounted dual-view laparoscopic imaging device with optical zoom and panning capabilities.

The current MRFL prototype (Fig. 2), software suite, and user interfacing are rich with features aimed at improving the safety and efficiency of MIS. This section provides an overview of these features and their respective aims. A full list of MRFL features and the MIS limitations they address is provided in Supplementary Table S2.

### MRFL design overview

The MRFL technology addresses a greater number of MIS safety and efficiency concerns than current alternative technologies while exhibiting fewer fundamental drawbacks due to the sagacious design which implements four key features: (1) simultaneous dual-view optical capture of a highly-magnified, narrow view and a less magnified wide-angled view (2) integration of electrically tunable lenses that enable optical zoom, autofocus, calibrated depth profiles, and manual fine focus for the zoomed view; (3) integration of a high-speed dual-axis scanning system that grants a surgeon or assistant the ability to pan the zoomed-view to any region visible within the wide view without physically moving the camera system; and (4) a custom, high-definition snub-nosed 10-mm diameter endoscope characterized by its short insertion length and extra-long counterpoised working distance (WD) aimed at minimizing clashing between the laparoscope and surgical instruments.

**Figure 3:**
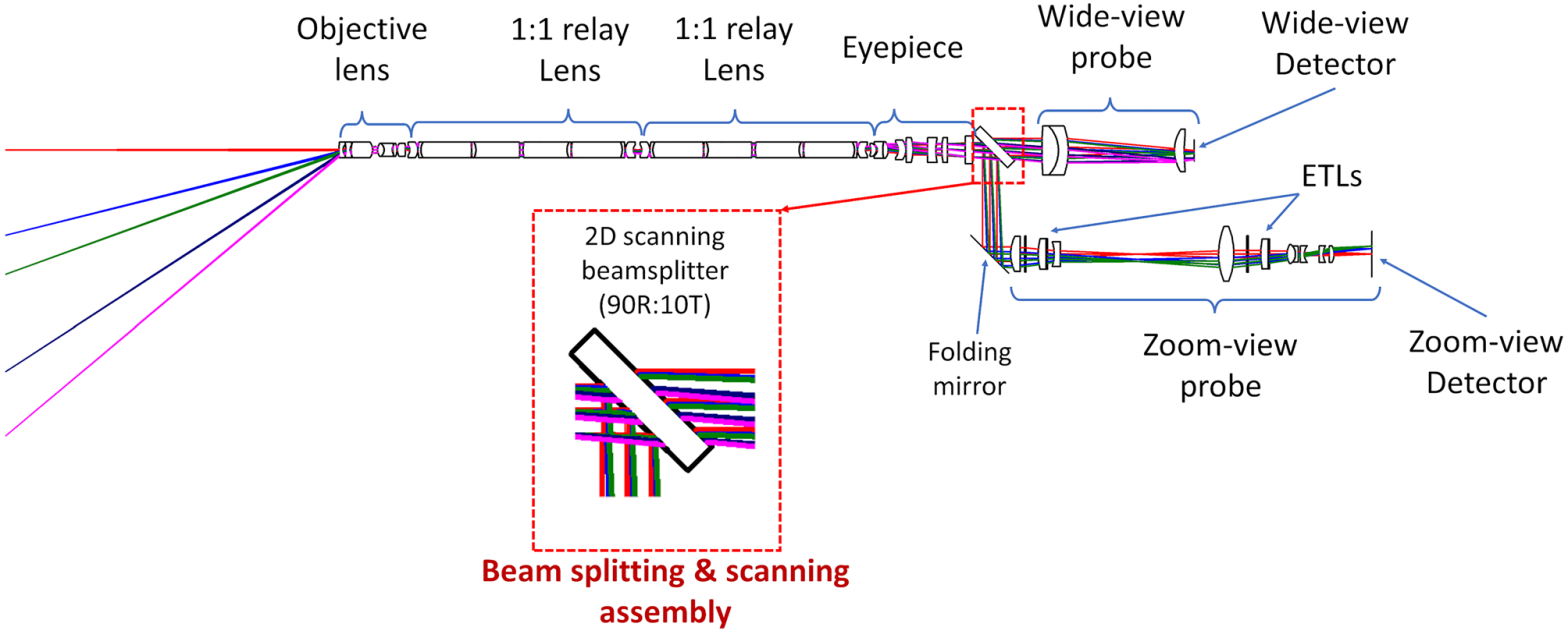
Optical layout of an MRFL prototype.

**Figure 4:**
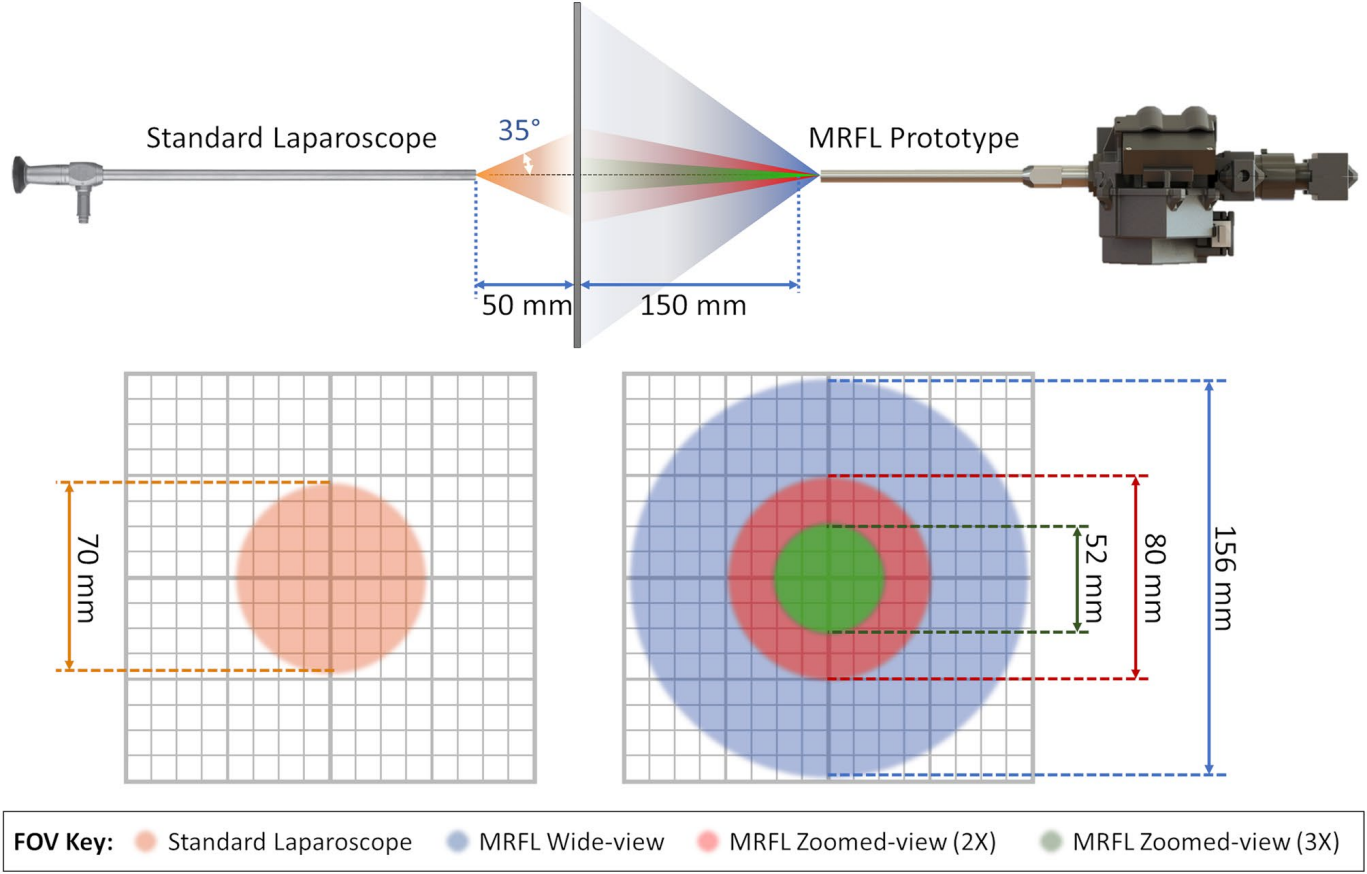
Diagram comparing the FOV and working distance of a standard laparoscope to that of the latest MRFL prototype.

The MRFL is a dual-view imaging device that captures and displays a wide and zoomed view of the surgical cavity simultaneously in real-time. The optical design layout of an MRFL prototype is pictured in Fig. 3. Unlike many related technologies that capture supplemental wide views through separate imaging devices, all light entering the MRFL is collected by a single set of endoscope optics comprising an objective and relay lenses. Following the endoscope optics are an eyepiece and beamsplitter that facilitate the systems panning capabilities and partition the light to two different imaging probes. The wide-view probe captures a large stadium-like view of the surgical cavity and the zoom-view probe images a narrow close-up view of the surgical ROI. Figure 4 illustrates the overlapping FOVs and WD of the wide-angle (the blue shaded area) and zoomed views (the red and green shaded areas for 2× and 3× zoom, respectively) for our most recent MRFL prototype in comparison to those of a standard laparoscope. The area imaged by the zoom-view probe at 2× zoom is comparable to that seen by a traditional laparoscope, while 3× zoom images a smaller area for finer details.

As illustrated in Fig. 4, the MRFL operates at working distances (the distance between the distal tip of the endoscope and the surgical ROI being imaged) between 120 and 150 mm, while a standard laparoscope operates at a WD of about 50 mm (or less) for close-up viewing. An extra-long WD facilitates the design of a snub-nosed endoscope with an intra-abdominal shaft length that is at least 100 mm shorter than a standard laparoscope. Thus, the MRFL offers a workspace between the ROI and laparoscope tip about three times larger than that of traditional laparoscopes. Since the laparoscope itself will always be outside the viewable area, the probability of undesirable instrument-laparoscope interactions, such as collisions and electrical coupling, increases as the laparoscope extends further into the body. The MRFL’s snub-nosed endoscope profile and large counterpoised WD lower the likelihood of unintentional intra-abdominal instrument-laparoscope interactions.

**Figure 5:**
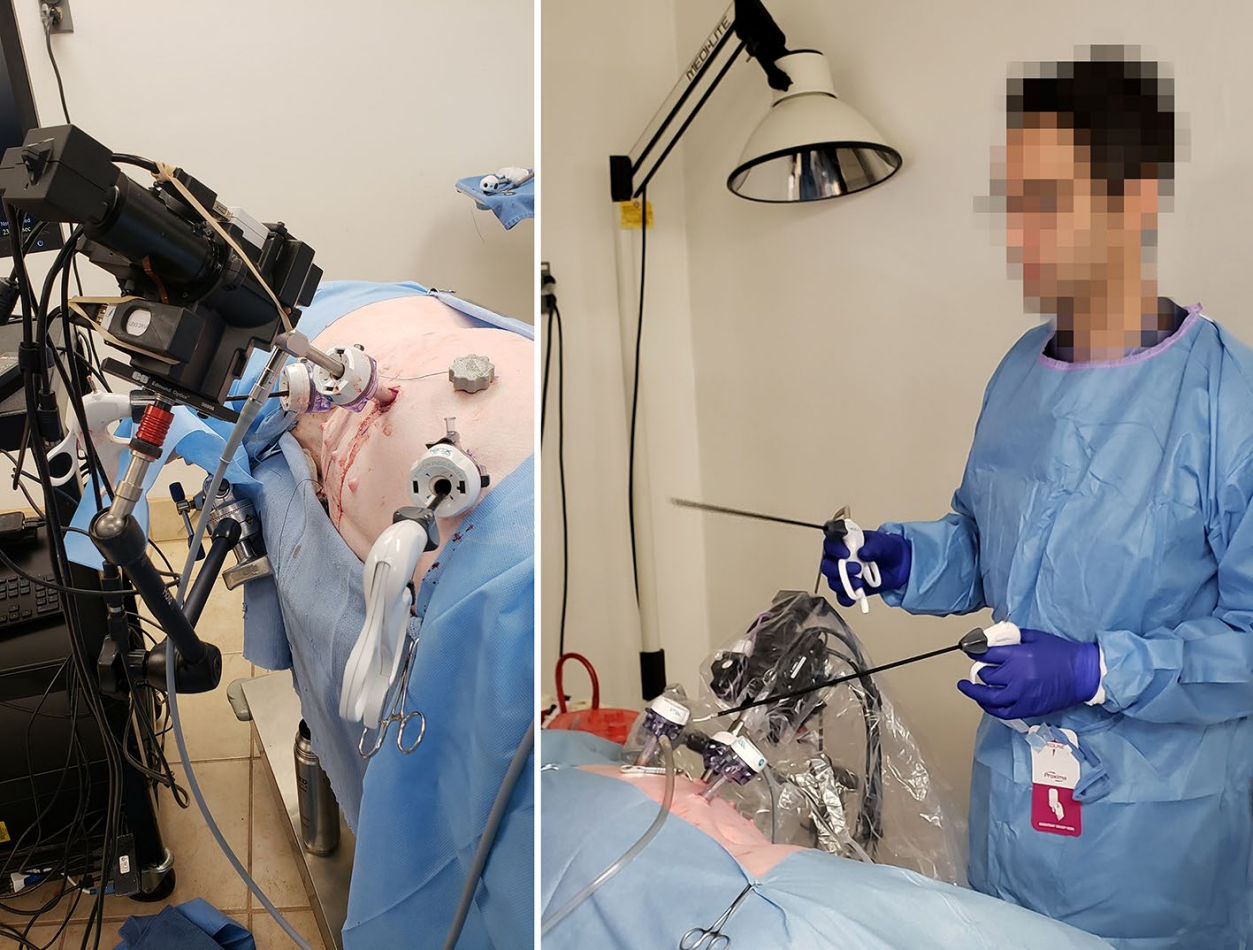
Photographs of the MRFL prototype being used for porcine surgery. The MRFL is mounted to the surgical table and remains stationary throughout the surgery (left). Thus, a surgeon may operate without need of an assistant in the immediate vicinity to hold the camera (right).

The MRFL system is designed to be kept at a fixed position and orientation throughout surgery as shown in Fig. 5. Two key imaging features are included in the design that allow the zoomed view to be manipulated in ways that mimic the imaging effects of physically repositioning a traditional laparoscope. Within the zoom-view probe are two electrically tunable lenses (ETLs) that change their focal powers based on the amount of electrical current sent through them. By varying the currents running through the tunable lenses, the zoom-view probe can zoom in and out on the surgical ROI resulting in the same imaging sensation experienced when advancing and withdrawing a traditional laparoscope. Furthermore, the beamsplitter is mounted in a motorized gimbal mount that allows for swift and precise dual-axis rotational control of the reflected light. By tilting the beamsplitter, the zoomed-view image can be panned to any ROI visible within the wide view, resulting in an imaging sensation similar to that experienced when pivoting a traditional laparoscope about its trocar fulcrum.

With these design features, we anticipate that a camera assistant would no longer be required to hold and manually operate the camera, as shown in Fig. 5. Consequently, ergonomic conflicts and hand-crossover situations common to standard hand-held laparoscopes are eliminated. Surgeons can potentially perform complex procedures more independently and efficiently with improved surgical precision due to their reduced fatigue and improved accuracy due to their increased ranges of motion.

### MRFL software and interfacing features

A custom software suite and user interface were created to facilitate and potentiate the MRFL design features discussed above as well as augment the system with additional capabilities to further address MIS safety and efficiency limitations. The software was designed to incorporate the following features: (1) a user interface that presents the dual-view information to users in an intuitive and beneficial format; (2) unique display modalities that accommodate a range of viewing preferences and an intuitive way to cycle through them; (3) a novel dual-monitor display interfacing that caters to the individualized needs of the surgeon and assistant; (4) a flexible controlling interface that allows the zoomed view to be manipulated by either surgeon or assistant and makes it possible for a surgeon to operate without an assistant in the immediate area; and (5) a hybrid method of automated and manual focus control that enables easy, quick, and accurate adjustment of the zoomed-view magnification and focus during surgery. These features are further explained as part of the system demonstrations in the following section.

## Demonstration of results and discussion

An MRFL prototype was constructed^39^ and tested in our laboratory using phantom imaging targets. Further testing was conducted by surgeons from Keck School of Medicine of USC in an in-vivo porcine surgical study. Surgeons that participated in the study saw great potential for the system to mitigate MIS safety and efficiency concerns but identified image quality as the prototype’s main drawback. A complete accounting of the Keck School study can be found in the cited literature^45^. This section demonstrates the MRFL system, software, and user interfacing features aimed at improving MIS safety and efficiency and briefly discusses the resulting applications and potential.

### MRFL system

**Figure 6:**
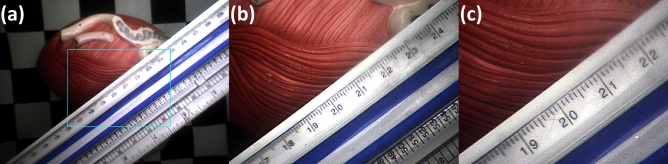
MRFL images used to measure the viewable area at the nominal working plane. The field of view was measured for the (**a**) wide-view, (**b**) 2× zoomed-view, and (**c**) 3× zoomed-view.

**Figure 7:**
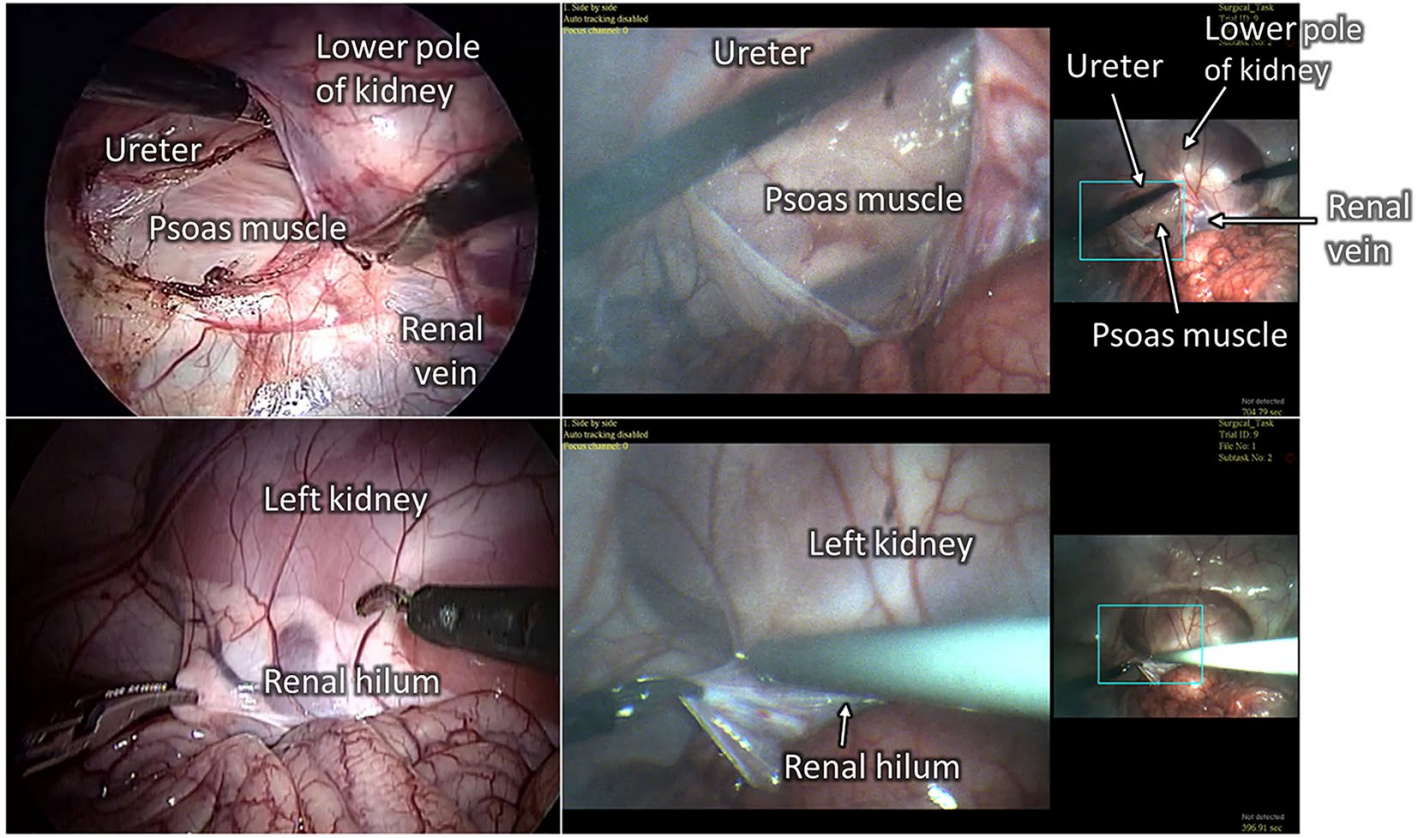
Images taken during surgical intervention. Images were captured using a standard laparoscope (left column) and the MRFL prototype (right column). The top row depicts lateral retraction of the lower pole of the right kidney. The bottom row demonstrates exposure of the hilar structures of the left kidney. The MRFL captures a close-up view similar to that of the standard laparoscope and a wide-angle view that enables visual monitoring of tools outside of the typical FOV (as seen in the upper-right MRFL image pair).

Figure 6a–c experimentally demonstrate the wide-angle, 2× zoomed view, and 3× zoomed view FOVs captured by an MRFL prototype, respectively. At the nominal WD of 120 mm, the FOV of the MRFL wide-view probe covers an area of 155.4 by 116.6 mm, while the zoomed view captures areas of 74.2 by 55.7 mm and 45.9 by 34.4 mm at 2 × and 3 × zoom, respectively. The measured average spatial resolutions for the wide view, 2×-zoomed view and 3×-zoomed view are 1.96 lp/mm, 3.86 lp/mm and 4.60 lp/mm, which parallel the average 2–5 lp/mm range exhibited by most high-definition traditional laparoscopes. Figure 7 depicts stages of porcine nephrectomies that were captured during in-vivo testing. For ease of comparison, each row of images depicts a similar stage of intervention as imaged by the Karl Storz Tricam camera head with a standard 10 mm diameter laparoscope (left) and the MRFL prototype (right). As shown in the figure, the MRFL successfully captures a zoomed view with field coverage similar to that of a standard laparoscope and a wide view with field coverage up to nine times larger.

**Figure 8:**
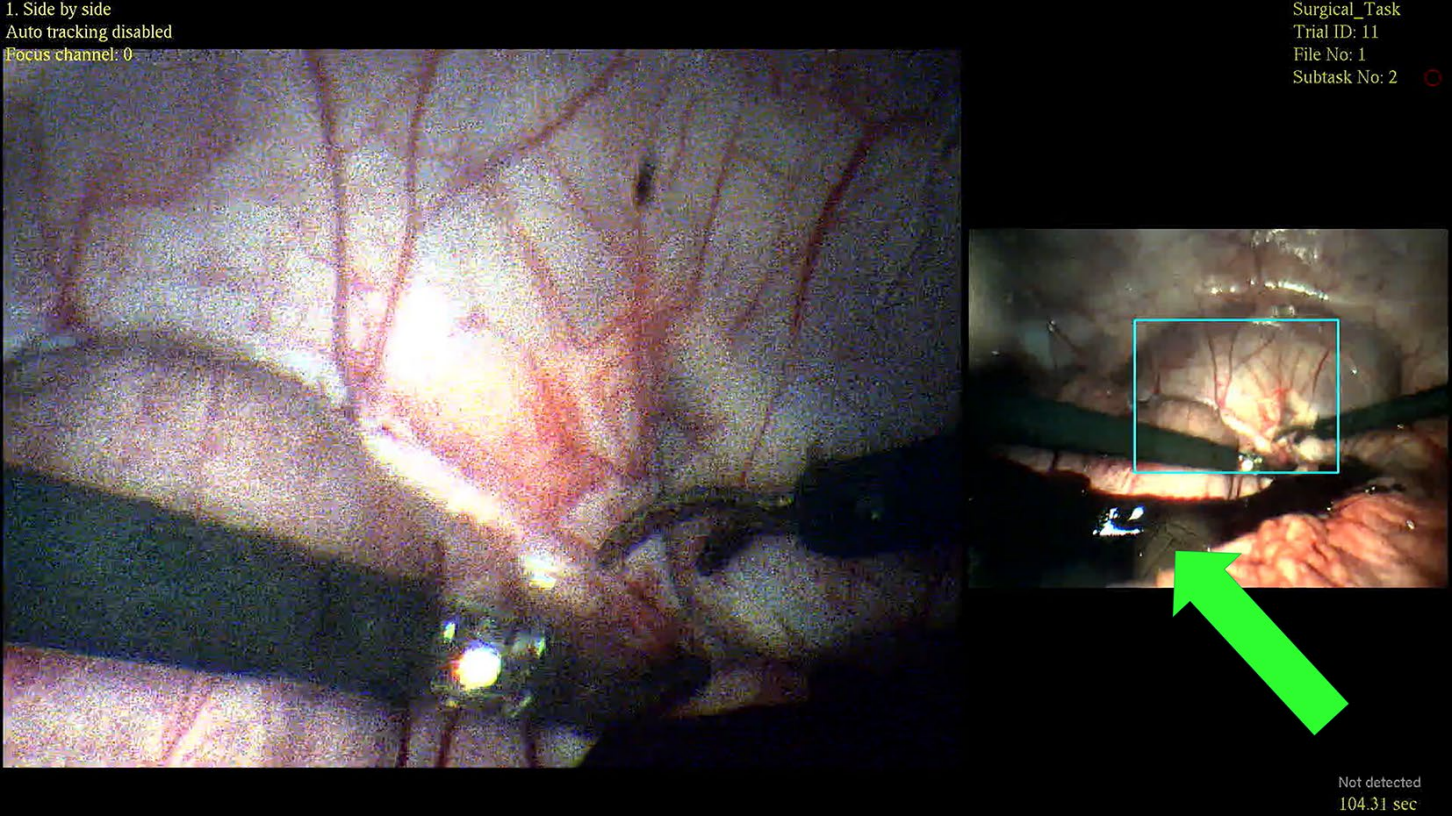
An image captured during in-vivo testing of the MRFL. Blood pooling from periphery is highly visible and easily seen in the wide view (green arrow) but not in the zoomed view. The dual-imaging nature of the MRFL allows for efficient visualization, prevention, and mitigation of adverse events in the field periphery.

Figure 8 shows an instance in which the dual views successfully augment situational awareness. Blood pooling in the periphery was visible and obvious in the wide view but not in the zoomed view. Given the similarity between the FOVs of the zoomed view and traditional laparoscope demonstrated in Fig. 7, such an event could also elude a commercial laparoscope. The real-time capture and display of a supplemental wide FOV to increase safety is a concept that has been explored before and found to be a viable and promising solution^46^. Providing a wide view and zoomed view simultaneously can reduce the number of incidents that go unnoticed in the periphery.

**Figure 9:**
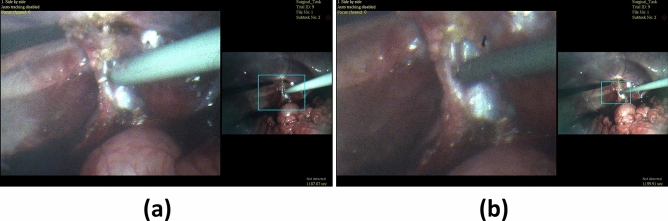
Images captured with the MRFL during porcine surgery. ETL autofocus ensures the zoomed-view image stays in focus through the full 2× (**a**) to 3× (**b**) zoom range.

**Figure 10:**
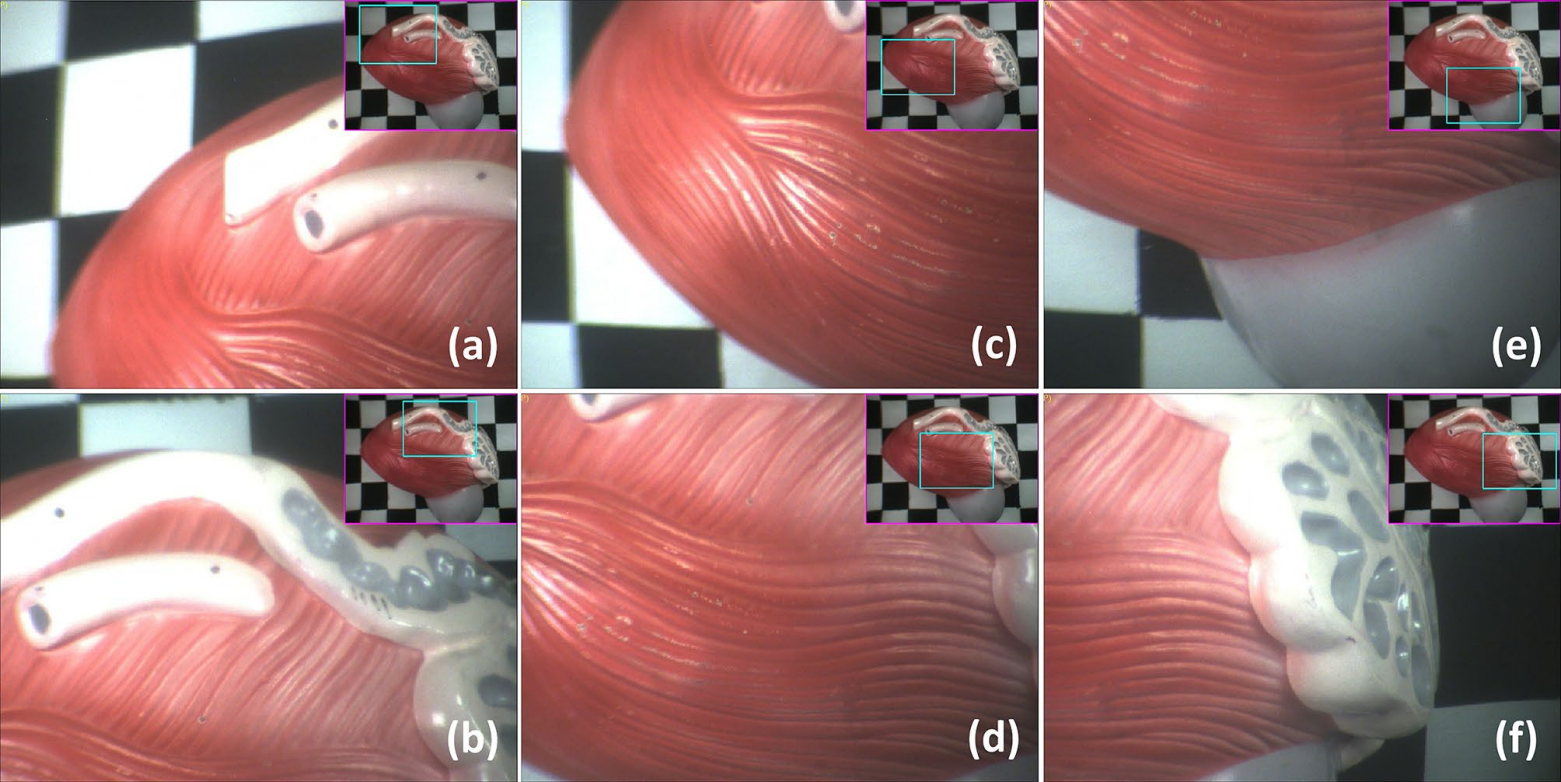
Demonstration of the zoomed-view panning capabilities using a urinary bladder and prostate model. The full model is visible in the wide view (upper-right-hand corner of each image set). Without moving the camera, the zoomed view was panned to descry all points of interest including the: (**a**) superior surface; (**b**) ampulla, ductus deferens, and ureter; (**c**) apex and anterior border; (**d**) inferolateral surface; (**e**) prostate base; and (**f**) seminal vesicle.

Additionally, the dual-view design successfully facilitates use of the MRFL as a stationary system. The large WD and wide-view probe allow the MRFL to capture a stadium-like view for situational awareness, which eliminates the need to withdraw the laparoscope during instrument insertion or other similar scenarios. Further, the zoom-view probe offers continuous control of optical zoom and autofocus through the integration of two ETLs. Adjusting the magnification of the zoomed view via the ETLs was found to be comparable to moving a standard laparoscope further from (Fig. 9a) or closer to (Fig. 9b) the ROI and thus eliminates the need for physically advancing the camera to gain higher spatial resolution. Similarly, the integrated high-speed dual-axis scanning system adequately eliminates the need for lateral repositioning of the camera by enabling the zoomed view to be rapidly steered to any ROI in surgical field that is visible in the wide view. The zoomed-view panning functionality is demonstrated in Fig. 10 and Supplementary Video S1.

### MRFL software and interfacing

**Figure 11:**
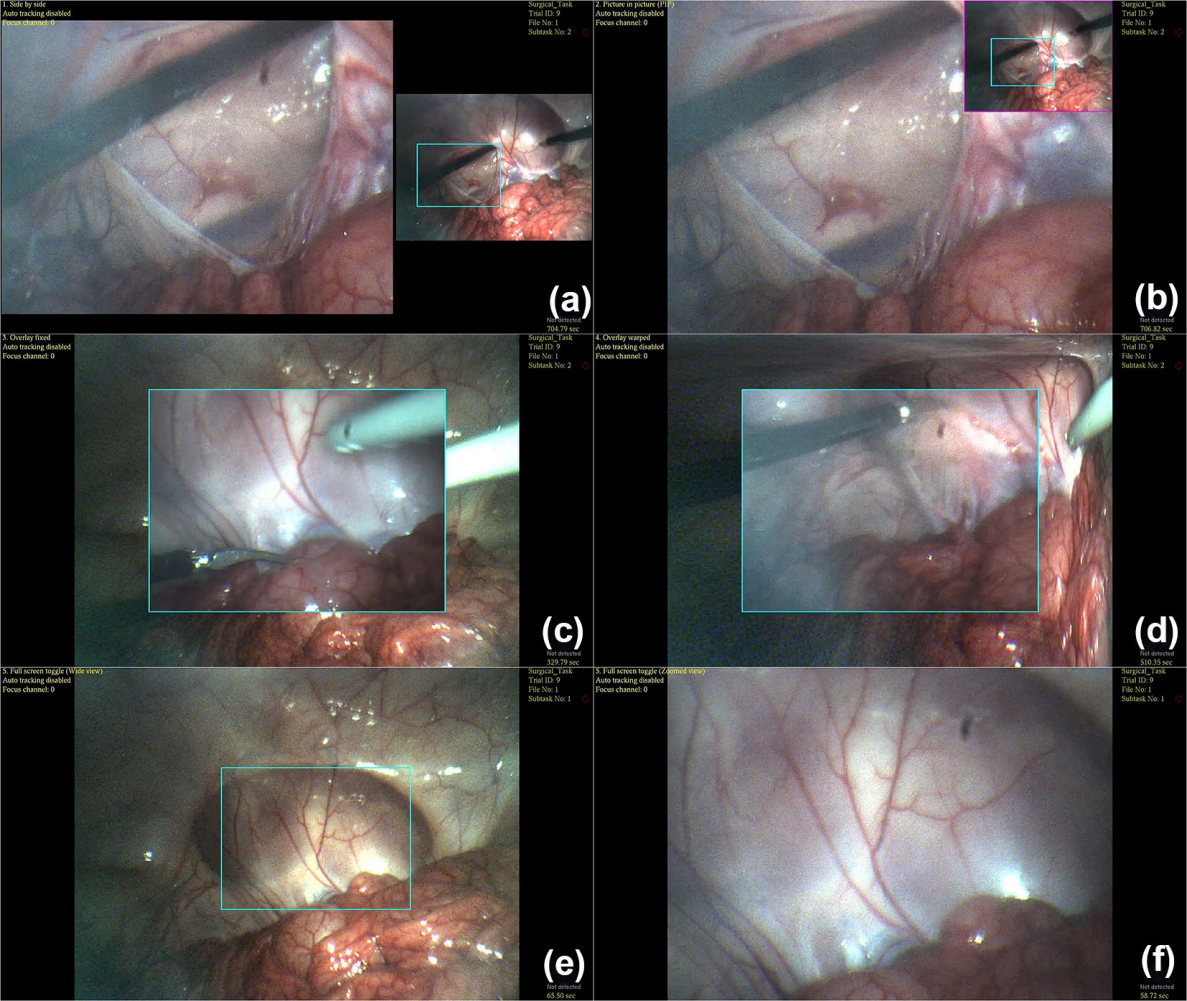
Images captured using different viewing modes during in-vivo testing of the MRFL. The view modes offered during in-vivo testing were: (**a**) O + D; (**b**) PIP; (**c**) FF + OC; (**d**) FF + WC; and (**e,f**) FST. These view modes are further demonstrated in Supplementary Video S1.

Depending on how the additional peripheral context is presented to users, simultaneous consumption and mental processing of the zoomed- and wide-view images can potentially increase a user’s cognitive load and stress. Therefore, the MRFL software suite is specifically designed to effectively present dual-view information to the user. As seen in the upper-righthand corner of each image in Fig. 10, the software overlays a cyan box in the wide view to indicate the zoomed-view position and FOV at any given time. Furthermore, the MRFL software provides up to eight different viewing modes that a user may cycle through according to their preference. Each viewing mode offers a unique format by which the wide-view and zoomed-view images are displayed. As demonstrated in Fig. 11, the available viewing modes (developed by Lee et al.^42,43^) include overview plus detail (O + D), picture in picture (PIP), focus plus occluded context (F + OC), fixed focus plus occluded context (FF + OC), focus plus warped context (F + WC), fixed focus plus warped context (FF + WC), focus plus unwarped context (F + UWC), and full-screen toggle (FST).

In the O + D mode (also known as the side-by-side mode), the wide-view and zoomed-view images are displayed in adjacent windows (see Fig. 11a). The PIP mode displays a large zoomed-view window and overlays a smaller window containing the wide-view in the upper right-hand corner (see Fig. 11b). The F + OC mode displays a large wide-view window and overlays a smaller zoomed-view window. The zoomed-view window moves around over the wide view offering a close-up view, much like a magnifying glass. Similarly, FF + OC (Fig. 11c) mode also overlays a smaller zoomed-view window over the larger wide view window. The zoomed view can still magnify any region within the wide view, but the zoomed-view window stays fixed in the center of the wide-view window. The F + WC mode is like the F + OC mode, except the wide-view image actively warps around the zoomed-view window to eliminate occlusion and maintain alignment of imaged features across the boundary between windows. Similarly, the FF + WC mode (Fig. 11d) is like the FF + OC mode, except the same warping effect is applied. In F + UWC mode, the wide view is displayed in full-screen with a smaller zoomed-view window overlayed. The low-resolution wide view is interpolated so that it matches the magnification of the zoomed view. The zoomed-view window moves around over the low-resolution wide-view image and displays a high-resolution view of the region over which it is positioned^42,43^. Lastly, FST mode (Fig. 11e,f) allows the user to toggle between full-screen versions of the wide view (Fig. 11e) and the zoomed view (Fig. 11f) via foot pedal or keypad.

**Figure 12:**
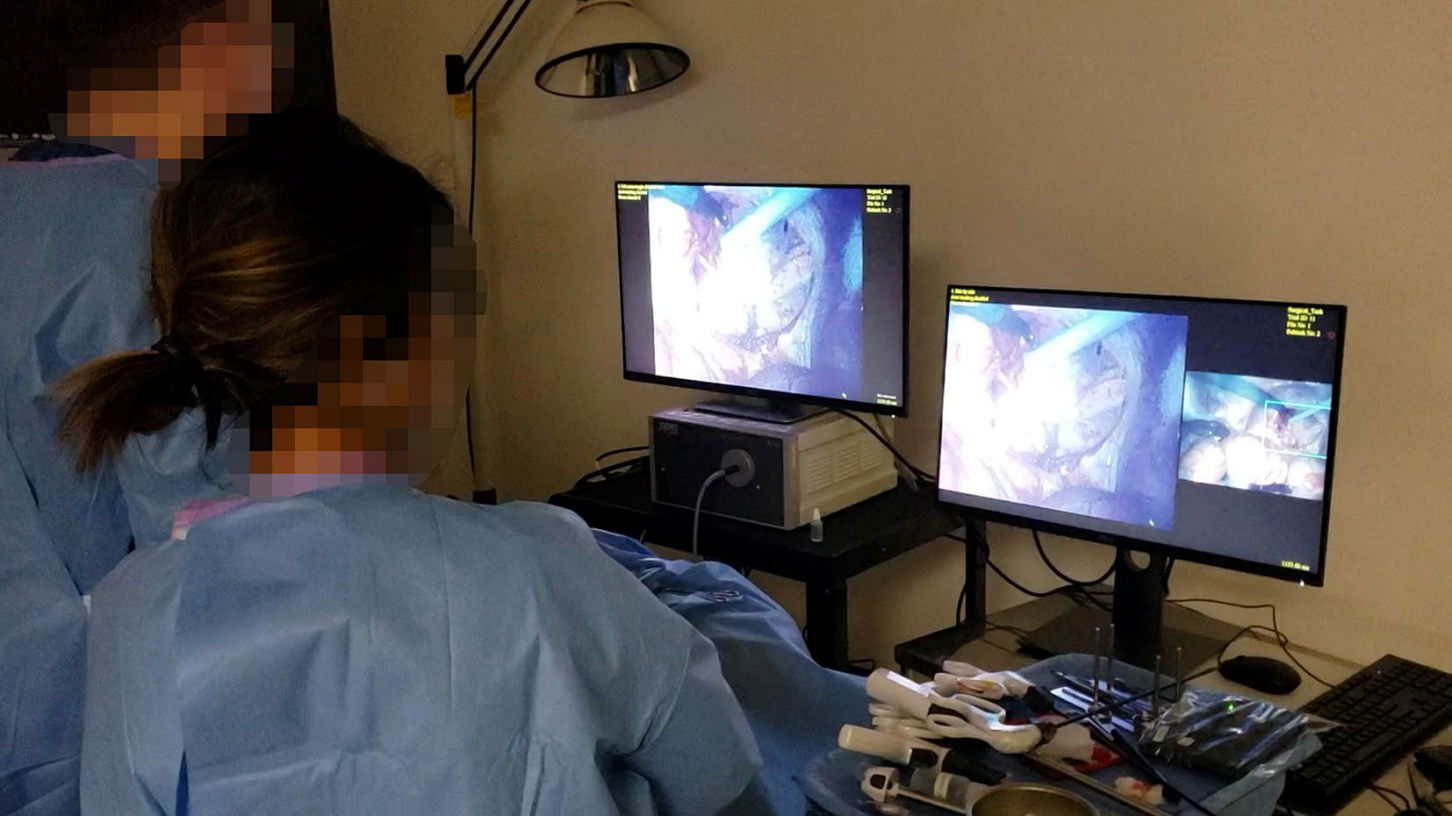
Photograph depicting the use of the MRFL with dual monitors during porcine surgery. The MRFL’s dual monitor feature allows a surgeon and assistant the ability to independently control the view mode displayed on their own monitor. In the instance captured above, the surgeon has opted for FST mode with the zoomed view set to full screen. The assistant, on the other hand, is using O + D mode. Either user may change their respective view mode at any time and as often as he or she sees fit.

The available display modes are designed to offer unique viewing experiences in accordance with our previous studies which suggest that display mode preference varies differently from user to user depending on the task being performed^42,43^. The tasks of a surgeon and assistant differ, so the MRFL software features dual-monitor functionality (shown in Fig. 12 and demonstrated in Supplementary Video S1) that provides a surgeon and assistant with separate monitors for which the viewing modes are independently controlled. Thus, the surgeon or assistant can toggle and control the viewing mode according to their preferences without affecting the other party. For example, the assistant may choose to switch their monitor to a viewing mode with a more prominent wide view of the surgical cavity while introducing an instrument into the patient. Meanwhile, the surgeon may continue operating unaffected.

The MRFL software suite offers manual and automated methods of controlling the dual-axis scanning system responsible for panning the zoomed view to any ROI within the surgical field. The manual method was developed for assistant use and allows the user to pan the zoomed view to the desired ROI via a keyboard interface without crowding the surgeon. Consequently, surgeons can potentially perform procedures more efficiently with improved precision and accuracy due to the increased range of motion.

The automated method allows selection of the desired ROI through an auto-tracking feature, which tracks the tip of an instrument in the wide view and repositions the zoomed view to the tool-tip’s location. This feature enables a surgeon to reposition the zoomed view quickly and intuitively just by pointing to the desired ROI with whatever instrument is currently being used. The MRFL offers two forms of auto-tracking, continuous and push-button, which may be engaged at any time via dedicated foot pedal. Continuous auto-tracking is activated by depressing and holding down the foot pedal and disengages when the pedal is released. While continuous auto-tracking is active, the zoomed-view will continuously follow the tool-tip around in a spotlight-like fashion. Push-button auto-tracking is more analogous to the point-and-click functionality of a computer mouse. The user places the tool-tip at the desired ROI and then triggers push-button tracking by quickly depressing and releasing the auto-tracking foot pedal. Once triggered, the zoomed view quickly repositions to image the indicated ROI. Both tracking methods are demonstrated in Supplementary Video S1.

**Figure 13:**
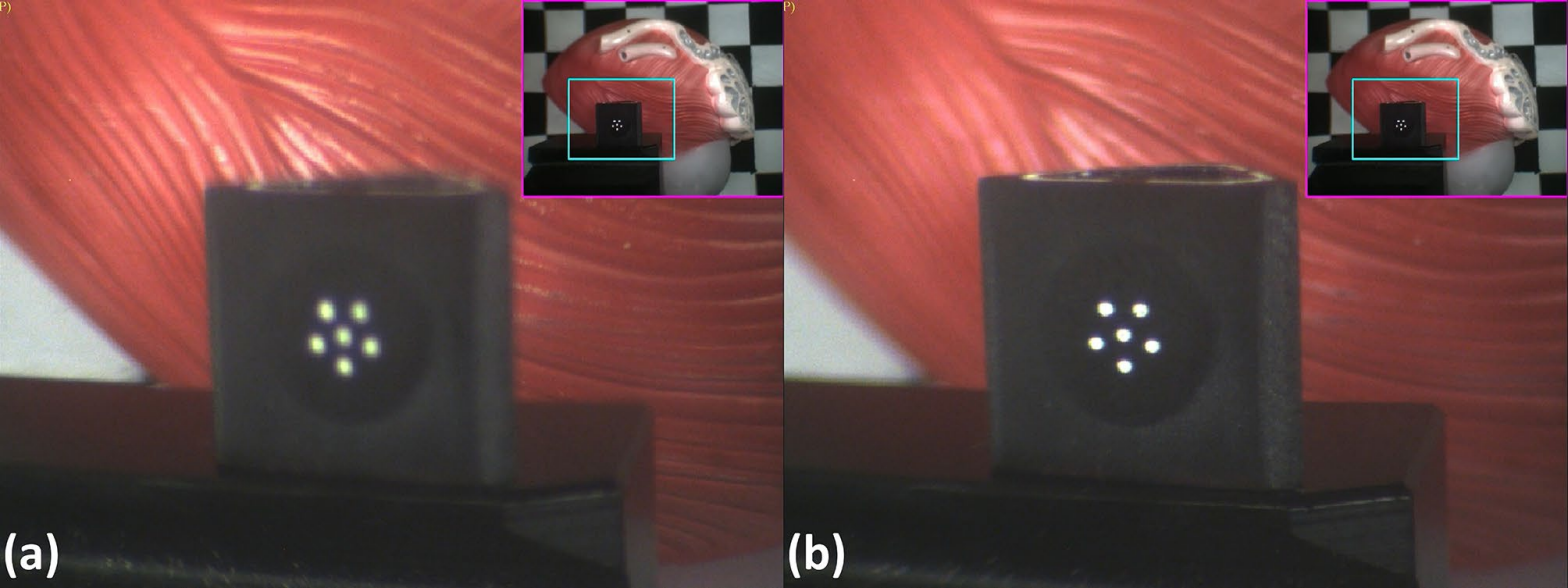
Demonstration of MRFL focus profiles. The profiles are calibrated at working distances of (**a**) 150 mm and (**b**) less than 50 mm. A bladder analogue and die were placed at those working distances respectively. The images shown were captured in quick succession without changing the setup or touching the camera. As intended, cycling through the focus profiles alternates focus between (**a**) the bladder analogue and (**b**) the die. This feature is also demonstrated in Supplementary Video S1.

Unlike the panning feature, zoomed-view magnification is currently only controllable via keyboard. The software suite, however, features autofocus capabilities with programmable focusing profiles for different WDs and manual fine-focus adjustment to ensure focus is easily achieved and maintained for any zoom factor. The programmable focus profiles allow the system to accommodate a large range of WDs (see Fig. 13 and Supplementary Video S1). Each focus profile can be configured to work at a specific WD and can be calibrated at distances outside the nominal WD range (120–150 mm) with the understanding that performance degrades accordingly. Additionally, the MRFL is equipped with a manual fine-focus adjustment to ensure that any point in the ROI is viewable in high detail to account for situations in which there is not a pre-calibrated focus profile that aligns perfectly with a sub-region of interest. It was found that combination of these features facilitates quick configuration of the MRFL camera before a procedure as well as simple adjustments during intervention, all without physically moving the camera.

### Limitations

The MRFL promises to be a safer, more efficient alternative to standard laparoscopes. Current limitations of the MRFL, however, must be overcome before it is considered as a viable, practical replacement. The current MRFL prototype was built as a proof of concept and thus is limited by its image quality, post processing hardware, auto-tracking method, packaging dimensions, and endoscope format.

The current system’s degraded image quality is attributed to the assembly process, low system throughput, and sensor inadequacy. Assembly complications and low system throughput arose as unforeseen products of the mechanical and optical designs, respectively. The inadequate detector choice, however, was deliberate since the purpose and budget of a proof-of-concept system does not warrant the purchase of a medical grade detector. Work has begun on the next generation of MRFL, a high-throughput multi-resolution foveated laparoscope (HT-MRFL), which features a medical grade detector and optical design that address the image quality limitations of the current prototype. Initial bench-top prototyping and testing of the HT-MRFL yielded superior imaging performance that is on par with commercial laparoscopes^47,48^, but a fully-functional, portable prototype fit for in-vivo testing is still years away.

The current MRFL image processing method is impractical and is not meant for clinical use. The video feed is passed from the cameras to a computer for post-processing before the final images are displayed on the monitor(s). Post-processing via external computer results in latency, unintuitive controls, and a cluttered operating room. Practical laparoscopic imaging systems are purpose-built devices with computer-like processing integrated into the camera and camera controller units. Currently, budget and development phase do not warrant integrating image-processing hardware and capabilities into a dedicated camera controller or into the MRFL itself. While in the research phase, a computer allows the needed freedom for development. Integration of processing into a controller, however, becomes a necessary step when transitioning from research and development to commercial production.

The auto-tracking capabilities of the current MRFL prototype are adequate for dry-lab testing, but the tracking method and algorithm are not robust enough for clinical use. The current tracking method involves two steps. First, a motion detection algorithm extracts the image of the tool from the wide-view image^49^. Second, a heuristic geometrical fitting is used to locate the tool-tip position. This form of auto tracking assumes that only one tool is present and that the tool is the only moving object within the wide view. Thus, the current system tracking falters in the presence of multiple tools or background movement, both of which are prevalent during MIS. Furthermore, the tracking algorithm is quite sensitive to specular reflections, which are commonplace during MIS due to the high-gloss surfaces of internal tissues, organs, and fluids. Improved auto tracking is a realistic and feasible goal for future MRFL systems. Numerous methods of auto tracking exist, but most fall into one of two categories, digital or physical. A third tracking category, hybrid tracking, exhibits a balanced compromise between the combined benefits and drawbacks of physical and digital tracking methods. A hybrid approach, such as that presented by Jung et al*.*^50^, would likely boost in-vivo tracking performance and could be easily adapted to work with the MRFL. Currently, we are considering methods from each category to improve the MRFL auto tracking capabilities.

At the time of design, only one commercial scanning mount offered the dual-axis scanning range, speed, and accuracy required for repositioning the zoomed view. The bulk of the system is attributed to the scanning unit and accompanying mechanisms. Since the fabrication of the current MRFL prototype, a compact scanning mirror has become commercially available that meets the system requirements. Thus, the next generation of MRFL will see a substantial reduction in size and weight^47^. Clinical use of the MRFL, however, requires that the mounting system be easily adjustable, steady, and facilitate reliable quick-release and reattachment of the MRFL if needed. Existing surgical rail mounting options are not adequate so robotic and counterbalanced armatures are being considered.

## Conclusion

This paper provides a detailed overview of the MRFL features designed to improve MIS safety and efficiency. The MRFL’s simultaneous capture of wide and zoomed views offers increased situational awareness that can improve MIS safety by decreasing the number of incidents that occur and go unrecognized outside the limited FOV of traditional laparoscopes. The static nature, dual monitor support, individualized view control and auto tracking capabilities of the MRFL can increase MIS efficiency by reducing fatigue and lessening dependence on surgeon-assistant interaction. The functionality of these features was validated and demonstrated through testing and phantom imaging. The limitations of the current MRFL prototype are image quality, bulk, auto-tracking, and computer-dependent processing. The next MRFL system, the HT-MRFL, accounts for image quality, bulk and auto-tracking, but it is not feasible at this time to transition to a computer-free processing alternative. In conclusion, the MRFL offers a more complete solution to contemporary MIS safety and efficiency limitations than competing technologies, and work is underway to increase its clinical practicality.

## Supplementary Information

Supplementary Tables.

Supplementary Video S1.

## Data Availability

No datasets were generated or analyzed during the current study.
