# Supplementary material for: A dual-view multi-resolution laparoscope for safer and more efficient minimally invasive surgery
Source: Sci Rep. 2022 Nov 2;12:18444. doi: 10.1038/s41598-022-23021-2 (PMC9630421; doi:10.1038/s41598-022-23021-2)
Supplement: Supplementary file 1 — Supplementary Tables. [file 41598_2022_23021_MOESM1_ESM.pdf]

Manuscript Title: A dual-view multi-resolution laparoscope for safer and more efficient minimally invasive surgery

Author List: Jeremy Katz[1], Hong Hua[1*], Sangyoon Lee[1], Mike Nguyen[2], and Allan Hamilton[3]

[1]James C. Wyant College of Optical Sciences, University of Arizona, Tucson, AZ, USA
[2]Department of Urology, Keck School of Medicine of USC, Los Angeles, CA, USA
[3]Division of Neurosurgery, Department of Surgery, University of Arizona, Tucson, AZ, USA

Supplementary Table S1:

**Significant Limitations**	**Augmented laparoscopes**	**CITs**	**IVNs**	**Current MRFL prototype**
Exhibits increased setup complexity	✗	O	✗	✓
Requires increased setup time	✗	✗	✗	O
Requires visual intracorporeal monitoring during setup	✗	✗	✗	✓
Withdrawal from the body exhibits increased complexity	O	O	O	✓
Withdrawal from the body requires increased time	O	O	O	✓
Requires visual intracorporeal monitoring during withdrawal	✓	O	O	✓
Poses sanitization or reusability concerns	O	O	✓	O
Subject to instantaneous FOV-resolution trade-off	O	O	✗	✓
Not a standalone solution (supplemental to a traditional laparoscope)	✓	✗	✗	✓
Rotational and translational disparities can occur between different supplemental views	✓	O	✗	✓
Rotational and translational disparities can occur between supplemental and laparoscope views	✓	✗	✗	✓
Increased intracorporeal footprint (as compared to a traditional laparoscope)	✗	✗	✗	✓
Handheld or requires physical movement	O	✗	O	✓
Requires multiple incisions or punctures	✓	O	✗	✓
Image quality impractical for clinical use	O	O	O	✗
Features intracorporeal innovations	✗	✗	✗	✓
Impractical insertable probe size	O	✓	O	✓

**Key**	
✗	Applies to most systems or is very problematic
O	Applies to some systems or is somewhat problematic
✓	Does not apply or is not a concern

Supplementary Table S2:

**Safety Concerns**		**Efficiency Concerns**	
**a**	Accidents occurring outside of narrow FOV	**g**	Entanglement and crowding from need for assistant to hold/maneuver camera
**b**	Incidents outside of FOV going unnoticed/untreated	**h**	Dependance on surgeon-assistant communication (especially for camera control)
**c**	Increased chance of **a** from blind tool insertion	**i**	Dependance on surgeon-assistant rapport
**d**	Collisions between endoscope and tissue/tools	**j**	Fatigue from holding camera
**e**	Non-ergonomic posturing to accommodate assistant holding/maneuvering the camera	**k**	Limited ranges of motion due to **e** and **g**
**f**	Loss of surgical horizon	**l**	Differing viewing needs between surgeon and assistant
		**m**	Trained Assistant is always required

**MRFL Feature**	**Concerns Addressed**	**Reasoning**
Low profile endoscope: short probe length and long working distance	**a, b**	Longer working distance allows greater FOV to be captured
	**d**	Allows larger region of space for instruments to move without the laparoscope being in the way
Dual-view: Simultaneous real-time capture of zoomed and wide views	**a, b**	A wide view of the surgical field can be visible at all times while using the zoomed-view for close-up detailed work
	**c**	The wide view can be used to guide tools to the surgical ROI during introduction
	**f**	The wide view provides orientational context that might otherwise be lost
	**h**	The wide view provides a contextual view that surgeon and assistant may reference to ease communication
	**l**	Dual-view capture facilitates the accommodation of a wider range of viewing needs
Multiple unique display modalities specifically designed for presenting dual-view information to the user	**a**	Constant peripheral visibility decreases chance of accidents occurring outside of the zoomed view
	**b**	Constant peripheral visibility increases chance of noticing accidents that occur outside of the zoomed view
	**c**	Constant peripheral visibility facilitates guided tool introduction
	**f**	Constant peripheral visibility facilitates visual monitoring of the surgical field orientation
	**l**	Simultaneous display of wide and zoomed views accommodates of a wider range of viewing needs and display mode can be chosen to best support current task
Zoomed view tool tip auto-tracking	**h, i, m**	Enables surgeon to directly control zoomed view panning without need for assistant
Zoomed view dual-axis panning	**b**	Zoomed view can easily be panned to address and treat incidents in the periphery
	**d**	Panning via scanning mirror eliminates need to move laparoscope thereby reducing risk of endoscope collisions
	**e, g, k**	Panning keyboard controls allow assistant to reposition zoomed view without crowding or interfering with surgeon
Adjustable optical zoom (zoom view probe)	**b**	Zoomed view can easily be zoomed in or out to tailor FOV to the incident being addressed or treated
	**d**	Zooming via ETLs eliminates need to move laparoscope thereby reducing risk of endoscope collisions
	**e, g, k**	Zooming keyboard controls allow assistant to adjust zoom without crowding or interfering with surgeon
Focus control: Auto focus, focus profiles, manual fine focus adjustment	**h, i, m**	Focus on surgical ROI is maintained without need for a trained assistant manually adjust the laparoscope
The system is designed to be mounted and stationary during surgery	**d**	Risk of endoscope collisions is reduced greatly since the MRFL system remains in a fixed position throughout surgery
	**e, g, h, i, j, k, m**	MRFL is mounted to the surgical bed railing so it does not require anyone to hold or physically maneuver it
	**f**	MRFL remains still throughout surgery so surgical horizon remains at a fixed position and orientation in wide view
Separate monitors for surgeon and assistant with independent viewing mode controls	**a, b**	Separate monitors for surgeon and assistant with differing view modes increases likelihood of incidents being noticed in the wide view and subsequently treated
	**c**	Assistant may easily change their view mode to visually monitor insertion without affecting surgeon
	**h, i, l**	Surgeon or assistant may switch viewing mode at will without having to anticipate how the other is affected
Keyboard controls for assistant	**e, g, k**	Panning and zooming keyboard controls allow assistant to manipulate zoomed view without crowding or interfering with surgeon
Dedicated foot pedal controls for surgeon	**g, h, i, k, m**	Foot pedals are ergonomic and allow surgeon to control many MRFL features without need for an assistant
	**l**	Foot pedal allows surgeon to switch the viewing mode on their monitor according to their needs without affecting the assistant
